# Quantitative single-cell analysis of immunofluorescence protein multiplex images illustrates biomarker spatial heterogeneity within breast cancer subtypes

**DOI:** 10.1186/s13058-021-01475-y

**Published:** 2021-12-18

**Authors:** Alison Min-Yan Cheung, Dan Wang, Kela Liu, Tyna Hope, Mayan Murray, Fiona Ginty, Sharon Nofech-Mozes, Anne Louise Martel, Martin Joel Yaffe

**Affiliations:** 1 Biomarker Imaging Research Lab (BIRL), Sunnybrook Research Institute, 2075 Bayview Avenue, Toronto, ON M4N 3M5 Canada; 2 Biosciences, GE Research (GER), Niskayuna, NY USA; 3 Department of Anatomic Pathology, Sunnybrook Health Sciences Centre, Toronto, ON Canada; 4 Department of Medical Biophysics, University of Toronto, Toronto, ON Canada

**Keywords:** Breast cancer subtypes, Biomarkers, Heterogeneity, Spatial arrangement, Protein multiplexing, Immunofluorescence imaging, Single cell imaging

## Abstract

**Background:**

The extent of cellular heterogeneity in breast cancer could have potential impact on diagnosis and long-term outcome. However, pathology evaluation is limited to biomarker immunohistochemical staining and morphology of the bulk cancer. Inter-cellular heterogeneity of biomarkers is not usually assessed. As an initial evaluation of the extent of breast cancer cellular heterogeneity, we conducted quantitative and spatial imaging of Estrogen Receptor (ER), Progesterone Receptor (PR), Epidermal Growth Factor Receptor-2 (HER2), Ki67, TP53, CDKN1A (P21/WAF1), CDKN2A (P16INK4A), CD8 and CD20 of a tissue microarray (TMA) representing subtypes defined by St. Gallen surrogate classification.

**Methods:**

Quantitative, single cell-based imaging was conducted using an Immunofluorescence protein multiplexing platform (MxIF) to study protein co-expression signatures and their spatial localization patterns. The range of MxIF intensity values of each protein marker was compared to the respective IHC score for the TMA core. Extent of heterogeneity in spatial neighborhoods was analyzed using co-occurrence matrix and Diversity Index measures.

**Results:**

On the 101 cores from 59 cases studied, diverse expression levels and distributions were observed in MxIF measures of ER and PR among the hormonal receptor-positive tumor cores. As expected, Luminal A-like cancers exhibit higher proportions of cell groups that co-express ER and PR, while Luminal B-like (HER2-negative) cancers were composed of ER+, PR- groups. Proliferating cells defined by Ki67 positivity were mainly found in groups with PR-negative cells. Triple-Negative Breast Cancer (TNBC) exhibited the highest proliferative fraction and incidence of abnormal P53 and P16 expression. Among the tumors exhibiting P53 overexpression by immunohistochemistry, a group of TNBC was found with much higher MxIF-measured P53 signal intensity compared to HER2+, Luminal B-like and other TNBC cases. Densities of CD8 and CD20 cells were highest in HER2+ cancers. Spatial analysis demonstrated variability in heterogeneity in cellular neighborhoods in the cancer and the tumor microenvironment.

**Conclusions:**

Protein marker multiplexing and quantitative image analysis demonstrated marked heterogeneity in protein co-expression signatures and cellular arrangement within each breast cancer subtype. These refined descriptors of biomarker expressions and spatial patterns could be valuable in the development of more informative tools to guide diagnosis and treatment.

**Supplementary Information:**

The online version contains supplementary material available at 10.1186/s13058-021-01475-y.

## Introduction

Breast Cancer is a highly heterogeneous disease. Methods to classify different clinicopathologic types of breast cancers range from evaluation of the immunohistochemical (IHC) staining of hormonal and protein biomarkers (Estrogen receptor ER, Progesterone receptor PR and Epidermal Growth Factor Receptor HER2/neu), etc., to molecular profiling and intrinsic subtyping based on RNA expression [1–3] and integrated transcriptomics with copy number aberrations (IntClust) [4–7] in bulk populations of cancer cells. Molecularly defined intrinsic subtypes classify breast cancers into Luminal A, Luminal B, HER2-enriched and basal-like cancers.

While these classification systems are clinically valuable for prognosis and treatment planning, the definitions of ER+/HER2-cancers as specified at the 2013 meeting of the St. Gallen International Breast Cancer Conference are quite broad [8] and display a spectrum of “intermediate” luminals (those between Luminal A-like and Luminal B-like) [9]. Discordance as high as 18% was found between clinical classification of hormonal receptor-positive and molecular subtyping of luminal cancers, with HR+ cancers often assigned to HER2-enriched and TNBC molecular subtypes, while clinically diagnosed HER2+ and TNBC included a fraction of Luminal cancers by PAM50 assessment [3, 10–17]. Detailed analysis of ER+ cancers with long-term outcome data has also identified integrative molecular subtypes that are associated with increased risk of long-term recurrence [18]. These findings suggest that additional specificity within this spectrum may be helpful in improving the characterization of this subtype. Heterogeneity was also observed in HER2+ and TNBC with impact on the response to treatment [15, 19–23]. The presence of inherent intra- and inter-tumoral heterogeneity could reduce effectiveness of treatment, possibly resulting in residual viable cancer cells which could contribute to resistance to treatment or recurrence [24–26]. Defining the cellular and molecular heterogeneities at the single cell level with spatial context will provide a more comprehensive understanding of breast cancer biology. Importantly, correlations between the molecular definitions of subtype with assays used in the clinical setting, namely morphology and IHC scoring, will help to translate these enhanced measures into functional tools for the clinical laboratory.

The purpose of this study is to assess heterogeneity within each clinical subtype of breast cancer in the context of protein marker expression signatures of single cells. Quantitative evaluation of the physical arrangement of cell subgroups is also illustrated to demonstrate the application of protein multiplexing in the investigation of spatial heterogeneities in breast cancer. In order to obtain a quantitative, single cell-based analysis of heterogeneity within breast cancer subtypes, we studied the co-expression patterns of breast biomarkers in individual cells of the cancer epithelium, the distribution of each subgroup and their spatial arrangement. Sequential staining of the same tissue section with a series of fluorescent-labeled antibodies, combined with digital imaging and bleaching of the fluorophores at the end of each staining round, was conducted using an immunofluorescence protein multiplexing platform MxIF [27]. In this study, we evaluated breast biomarkers ER, PR, HER2 and Ki67, together with TP53, CDKN2A (P16INK4a), cyclin-dependent kinase (CDK) inhibitor CDKN1A (P21/WAF1) in individual cancer cells of a breast tissue microarray (TMA) comprising invasive tumors. Subgroups of cells based on ER, PR, HER2 and Ki67 MxIF positivity were identified, and their abundances and spatial distributions were studied and compared between the pathologic IHC-based subtypes. Co-expression patterns of TP53 and P16 were also studied. In addition, CD8 and CD20 lymphocytes in the tumor microenvironment were quantified and their spatial localization was analyzed.

## Methods and materials

### Breast cancer tissue microarray (TMA)

The formalin-fixed, paraffin-embedded (FFPE) breast cancer TMA used in this work was obtained from Pantomics (CA, USA). It includes 75 cases of benign tissue and invasive breast cancer with two independent cores (1.1 mm diameter) taken from different regions of the same specimen (referred as paired cores in this study). Only cores with invasive cancers were analyzed.

### Immunohistochemical staining

Serial sections of the TMA were stained with protein marker in immunohistochemistry either using the Ventana autostainer (Roche), or manually, following manufacturers’ protocols. The following antibodies were used: Estrogen Receptor (SP1, Ventana Medical Systems), Progesterone Receptor (1E2, Ventana Medical Systems), HER2 (4B5, Ventana Medical Systems), Ki67 (MIB-1, Ventana Medical Systems), P53 (DO-7, Dako), P16 (CINtec, Ventana Medical Systems), P21 (1:200, Cell Signaling Cat#2947). ER, PR, HER2 and P21 IHC were scored by study pathologist (K.L.) with an evaluation of staining intensity and percentage of positive cells [28]. HER2 scoring was conducted according to the recommendations from 2013 ASCO/CAP [29]. HER2 2+ (equivocal) cases by IHC were further evaluated with fluorescent in situ hybridization (FISH) (S. N-M). Ki67 IHC was presented as percentages of positive cells per core (scored by K.L.). P53 and P16 IHC were reviewed by study pathologist (S. N-M.) and categorized as normal, null or over-expressed for P53 and normal or over-expressed for P16.

### Classification of intrinsic subtypes based on IHC surrogate staining

Based on scoring of ER, PR, HER2 and Ki67 IHC of serial sections of the TMA, cores were classified as Luminal A-like [LumA], Luminal B-like (HER2-negative) [LumB], Luminal B-like (HER2-positive) [LumB, HER2 +], HER2+ (non-luminal) [HER2 +] and TNBC [TNBC] based on criteria described in Goldhirsch et. al. [30]. Of the TMA cores studied, 24 (14 cases) were LumA cores, 36 (21 cases) were LumB cores. One case has one core classified as LumA, and the second core classified as LumB. Six cores (4 cases) were LumB, HER2+, 13 cores (9 cases) were from HER2+ cancers, and 22 cores (12 cases) were from TNBC.

### Antibody selection, conjugation, calibration and validation of MxIF

Commercially available antibodies for each protein marker of interest were first tested using standard IHC with appropriate positive and negative control tissues. Antibodies that showed specific staining and intensity comparable to IHC were selected and conjugated to Cy3 or Cy5 fluorophores following established protocols [27]. Each fluorophore-conjugated antibody was optimized and validated using control tissues. The staining patterns, specificities and intensities of conjugated antibodies were compared to the optimized IHC staining of the same tissue and evaluated by study pathologists (K.L, S.N-M). For breast biomarkers of ER, PR, HER2 and Ki67, we used clinically annotated breast cancer tissues for antibody validation. P53 staining was validated with a colon cancer case demonstrating P53 overexpression, while P21 and P16 were validated with skin and colon tissues and on high-grade serous ovarian cancer cases, respectively. A range of concentrations were tested for each fluorophore-conjugated antibody in the MxIF staining protocol and the parameters that resulted in staining intensities similar to those with IHC as performed in the clinical immunohistochemistry lab were selected.

### Protein multiplexing

Protein multiplexing was conducted on the Immunofluorescence Multiplexing MxIF platform (GE Research (GER), Niskayuna, NY, USA) [27]. Pairs of fluorophore-conjugated (Cy3 and Cy5) antibodies were sequentially applied onto a single tissue section of the TMA, followed by image acquisition and photo-induced chemical bleaching to inactivate optical signals from antibodies (U.S. patent 7,741,045) [31]. The order of antibody staining is listed in Table 1. In the first round of staining, unconjugated antibodies for HER2 and P16 were applied in primary incubation, followed by secondary staining with fluorophore conjugates of Cy3 or Cy5, respectively. All other antibodies were directly conjugated to fluorophores. After completion of MxIF staining rounds, quality of the staining of each protein marker was visually evaluated. TMA cores showing non-specific staining or having high background, over-exposed areas, or exhibiting tissue damage were excluded from analysis. Antibodies for the cyclooxygenase-II enzyme (COX-2) were included in the panel and data will be presented in a future report.

**Table 1:** List of antibodies, fluorophore conjugated to each, and the staining sequence in MxIF. Na^+^K^+^ATPase and Ribosomal S6 were used as segmentation markers for membrane and cytosol, respectively. Pan-cytokeratin (PCK26) was used as the epithelial cell marker. COX2 was also included in the staining rounds but data not analyzed in the current study

Staining round	Cy3				Cy5			
	Antibody (clone)	Supplier	Cat #	Unconj or Conj	Antibody	Supplier	Cat #	Unconj or Conj
1	HER2/neu (SP3)	ThermoFisher	RM9103	Unconj	p16 (CINtec)	Roche/Ventana	725–413	Unconj
2	Na^+^K^+^ATPase	Abcam	ab167390	Conj	KI67(SP6)	Zeta	Z2031	Conj
3	PR	Dako	M3568	Conj	ER(SP1)	Spring Bioscience	M3014C	Conj
4	Ribosomal S6	Cell Signaling	2217BF	Conj	p21	Cell Signaling	2947BF	Conj
5	PCK26	Sigma	C5992	Conj	p53	Dako	M7001	Conj
6	–				COX2	ThermoFisher	187379	Conj
7	–				CD20	Abcam	ab166865	Conj
8	–				CD8	Dako	M7103	Conj

### Image analysis and statistical methods

Image registration and single cell segmentation were performed with the software package, Layers, developed by GER for the MxIF platform. The DAPI signal and those from antibodies of ribosomal S6 and Na^+^-K^+^-ATPase were used for segmentation of the cell nuclear, cytoplasmic and membranous compartments, respectively, as well as the boundary of individual cells. *Layers* uses one channel to highlight the nuclear component and a second to represent intracellular membranes, extracellular fibers and other tissue structures, to produce a “virtual H&E” (vH&E) image. Following cell segmentation, measurements of cell size, morphology and protein marker signals were collected together with the coordinates of cell centroids. Pre-processing of data was conducted to remove cells that did not meet quality control requirements (i.e. cells that were lost or damaged at some point in the processing procedure). Pixel size in our study was 0.293 μm.

### Inclusion of cancer cells for analysis

For the study of the cancer population, a set of cell size parameters (> 90 pixels in perimeter, > 400 pixels in cell area and > 10 pixels of nuclear area) were applied in gating and included for analysis. To enrich for cancer cells in our analysis, single cell data from Luminal A/B and HER2+ cores were gated with Pan-Cytokeratin (PCK26) level (> = 1500 raw signal intensity) in order to select for PCK+ epithelial cancer cells. Regions with benign ducts were manually annotated by study pathologist (K.L.) on the virtual H&E images using the Image viewer Sedeen (Pathcore Inc, Toronto, ON). A python script (http://www.python.org) was developed to include/exclude single cell data from annotated regions. In this case, single cell data corresponding to the annotated benign ducts were excluded from analysis. For TNBC, PCK staining is generally weak and prevented gating for PCK+ epithelial cells. Subsequently, cancer cells were manually annotated using Sedeen and the python script was applied to select for cells in the annotated regions.

### Data processing and thresholding

Protein expression levels for each marker were extracted from their reported subcellular compartment localizations as the fluorescent marker pixel values (ER-nuclear, PR-nuclear, HER2-membranous, Ki67-nuclear, P53-nuclear and cytosol, P21-nuclear, P16-nuclear, PCK-membranous). A min–max normalization to a range of 0–15 was performed on the values for each marker individually. For thresholding analysis of ER, PR and HER2, selection of empirical cut-points for marker positivity (weak vs strong staining for ER) were determined by comparing the MxIF values of cells from cores that were scored as negative, weak, moderate or strong staining intensities on IHC. For HER2, MxIF values were compared to cores scored as 0, 1+, 3+ in IHC as cases with equivocal (2+) scoring were further tested with FISH. Five cores in each scoring class were studied (3 cores only for PR weak). For Ki67, the cut-point was determined by comparing MxIF distributions from representative cores with a range of IHC scores. For P53, P16 and P21, cut-points were determined at > 0, 2 and 1 of normalized MxIF levels, respectively, after comparison with IHC scores.

### Immune cells analysis

All segmented cells (including cancer and stromal cells) were included for analysis. CD8-positive and CD20-positive cells were quantified based on a threshold setting of normalized MxIF signals at 0.2.

### Spatial arrangement of single cells

A MATLAB script was developed to map and label individual cells with their protein marker classifications using their cell centroid coordinates. Neighborhood analysis was conducted by calculating the frequency of each neighboring cell type within a 30 μm (100 pixel) or 100 μm (341 pixel) radius from the centroid of the central cell. Heterogeneity was evaluated by calculating the Shannon Equitability index (E_H_) (Shannon Diversity Index divided by maximum diversity defined as [Ln (Number of Species)]) [32]. It assumes a value between 0 and 1, with 1 representing the condition of all species present in the neighborhood and at the same level of abundance.

### Statistical analysis

Significant difference between each pair of cores taken from the same cancer specimen was determined with the compare.2.vectors function (afex package in R) with the EPH and Ki67 classifications (16 classes) for all cells in each TMA core presented as a vector. *p* < 0.01 for both Wilcoxon test (coin::Wilcoxon) and permutation test were used to determine significant difference. Significant differences between the percentages of cells in each EPH group among IHC subtypes, and between E_H_ of EPH groups were evaluated with ANOVA test and Tukey’s HSD. All statistical analysis were performed in R (R Statistics).

## Results

### MxIF intensities of biomarkers in single cells showed distributions comparable to IHC scoring of tissue core

**Fig. 1:**
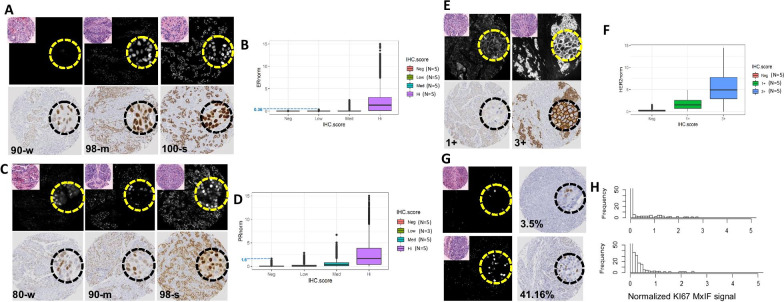
Comparison of MxIF biomarker signal intensities to IHC scoring on serial sections. **A**, **C**, **E** Paired MxIF with vH&E in inset (top) and IHC (bottom) images of ER (**A**), PR (**C**) and HER2 (**E**) of representative cores are shown. A small region of each image is zoomed in 40 × magnification (dotted circle). The corresponding IHC score for each core is as indicated. For ER and PR, IHC were scored with the percentage of positive cells and average marker intensity. For example, 90-w indicates 90% positive cells with weak staining. m-Moderate staining, s- Strong staining. **B**, **D**, **F** The distribution of normalized MxIF signal intensities from single cells in cores that were classified based on their corresponding IHC staining on serial sections for ER (**B**), PR (**D**) and HER2 (**F**). Normalized MxIF values (ERnorm, PRnorm or HER2norm) from single cells were compared between cores where IHC scoring indicated that they are either negative (Neg), or a majority (60% +) of cells were scored as weak (Low), moderate (Med), or strong (High) for ER and PR, and negative, 1+, 3+ for HER2. The number of cores (N) included in each group is as shown. **G** Ki67 comparison. Paired MxIF and IHC of representative cores with the indicated IHC scores of positive percentages are shown. **H** Distribution of normalized Ki67 MxIF signal intensities from the same cores

A total of 225,086 single cells from 101 TMA cores (59 cases) were included after quality evaluation and gating parameters had been applied (see Additional file 1). From the 101 TMA cores studied, 82 were paired cores from 41 cases. In order to determine if MxIF measurements of breast biomarkers reflect IHC results, we compared the distribution of normalized ER and PR MxIF signals from individual cells between cores that were scored by IHC as negative, weak, moderately or strongly stained for the majority of the cells (Fig. 1A–D). For HER2, the distributions of normalized MxIF signals from individual cells were compared between cores that were scored in HER2 IHC as negative, 1+ or 3+ (2 cases that were scored as 2+/equivocal in IHC were further tested by FISH with one case re-classified to 1+ and one to 3 +)(Fig. 1E, F). We found that ER MxIF signal intensities were relatively low in cores scored as weak or moderate by IHC and that there was significant overlap in the range of MxIF values in those cores (Fig. 1B). Some cells in cores that were scored as strong in IHC also exhibited zero or low MxIF signal intensities, but the majority of cells showed medium to high MxIF staining intensities. For PR, MxIF signal intensities for negative and weakly stained cores again showed overlapping distributions, with cells from moderate and strongly stained IHC cores showing higher intensities and more dispersed range (Fig. 1D). MxIF HER2 staining showed discrete distributions between neg (0), 1+ and 3+ IHC-scored cores (Fig. 1F).

Based on the distributions of normalized MxIF intensities for each protein marker, threshold levels were determined for binary classification of cells. As the maximum MxIF level of cells from weak ER IHC cores (IHC score = Low) was 0.36 (Fig. 1B), a threshold of 0.4 was chosen to segregate cells with negative or weak staining from the moderate and strongly positive cells. The cut-point to classify PR positive and negative cells was set at 1.6 as the range of normalized MxIF cell intensities from IHC PR-negative cores extended from 0 to 1.6 (Fig. 1D). The HER2-positive cut-point was set at 5.0, the maximum signal intensity from cells in HER2 1+ cores (Fig. 1F). Comparison of representative cores with various levels of Ki67 IHC positivity prompted the selection of a cut-point value at 0.2 to include the weakly positive cells (Fig. 1G, H).

**Fig. 2:**
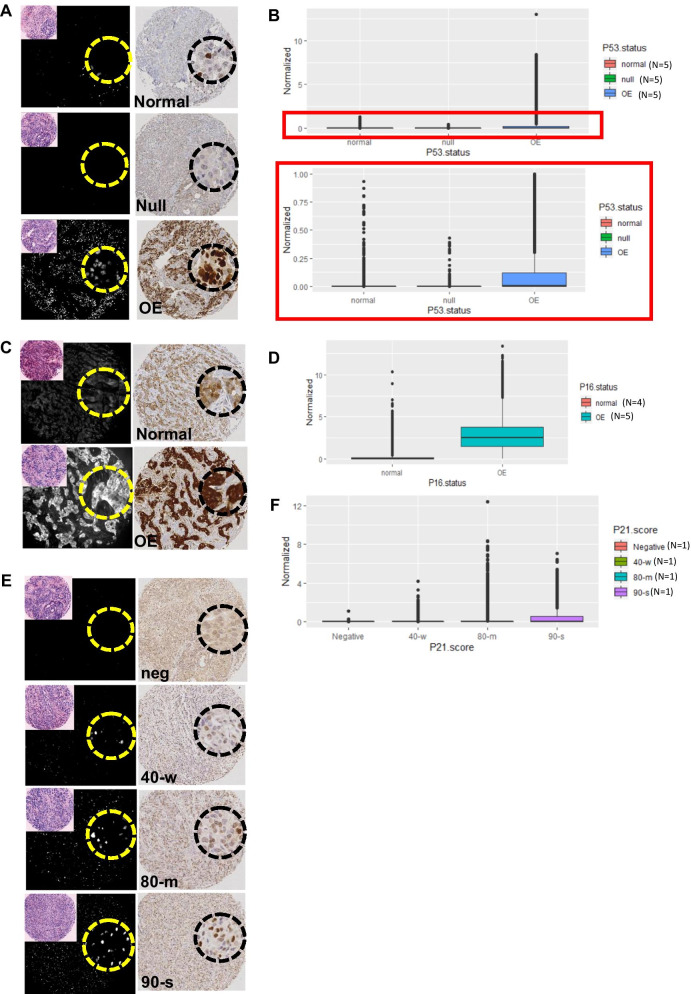
Distribution of MxIF signal intensities of P53, P16 and P21 from cores with various IHC patterns. **A** Comparison of P53 staining pattern from MxIF and IHC of representative cores which were classified as normal, null or overexpressed (OE) with IHC scoring. Virtual H&E image of the corresponding core in MxIF is shown in inset. A small region of each image was zoomed in 40 × magnification (dotted circle). **B** Boxplot graphs showing MxIF P53 normalized signals from single cells in cores classified as P53 normal, null or overexpressed (OE) with IHC evaluation (top). The plot is further magnified (bottom). The number of cores (N) included in each group is as indicated. **C** P16 evaluation of MxIF and IHC staining of cores that were classified as normal or overexpressed (OE) in IHC. **D** Boxplot graphs showing MxIF P16 normalized signals from single cells in cores that were classified as normal or overexpressing (OE) P16. **E** P21 IHC were scored with the percentage of positive cells and average intensity. Representative cores with negative staining (neg), 40% weak (40-w), 80% moderate (80-m) and 90% strong (90-s) are shown, together with MxIF staining of the same core. **F** Boxplot graphs showing P21 MxIF normalized signals from single cells in cores with the indicated IHC scores

In addition to ER, PR, HER2 and Ki67, we also used MxIF to study the expressions of P53, P21 and P16 on the TMA. Pathologists evaluate P53 and P16 IHC staining in cancers by reviewing the overall staining intensity and pattern across the lesion and classify tumors as normal (heterogeneous staining across the lesion), null or overexpression for P53 and normal or overexpression for P16 [33, 34]. We studied the distribution of MxIF signal intensity levels of each marker in cores that showed various IHC staining patterns (Fig. 2). P53 MxIF signals in cores that were scored by IHC as null ranged from 0–0.5 (Fig. 2A, B). P53 normal cores expressed a majority of cells with P53 MxIF at 0, with some cells expressing levels between 0 and 1, whereas some cells in cores with overexpression (O/E) by IHC exhibited much higher levels of MxIF signal intensity (99^th^ percentile at 6.77). In a comparison of P16 MxIF levels between IHC normal or O/E cores, cells from O/E cores tended to exhibit higher measured intensity than normal cores (Fig. 2C, D). P21 expression in IHC was scored in terms of percentage of positive cells and average staining intensity. P21 MxIF values from four cases with varying IHC scores were compared (Fig. 2E, F). MxIF values from the P21 negative core remained low at 0 to 1. As the percentages of IHC positive cells and their staining intensities increased, a corresponding increasing trend in MxIF signal intensity was observed. These data suggest that MxIF signal distributions could be used to reflect the mutational status of P53 and P16 or P21 expression patterns determined by IHC evaluation.

### MxIF co-expression pattern of ER, PR, HER2 and Ki67 recapitulates IHC-surrogate subtypes and identifies heterogeneity within each subtype

**Fig. 3:**
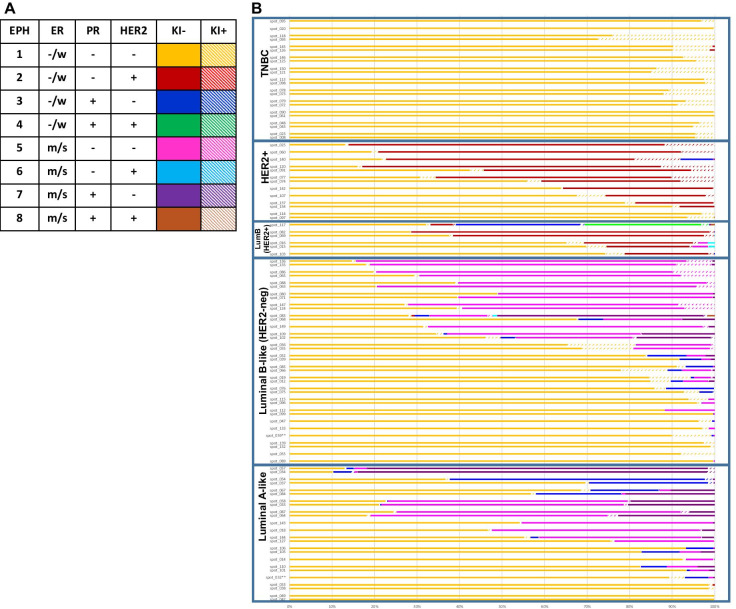

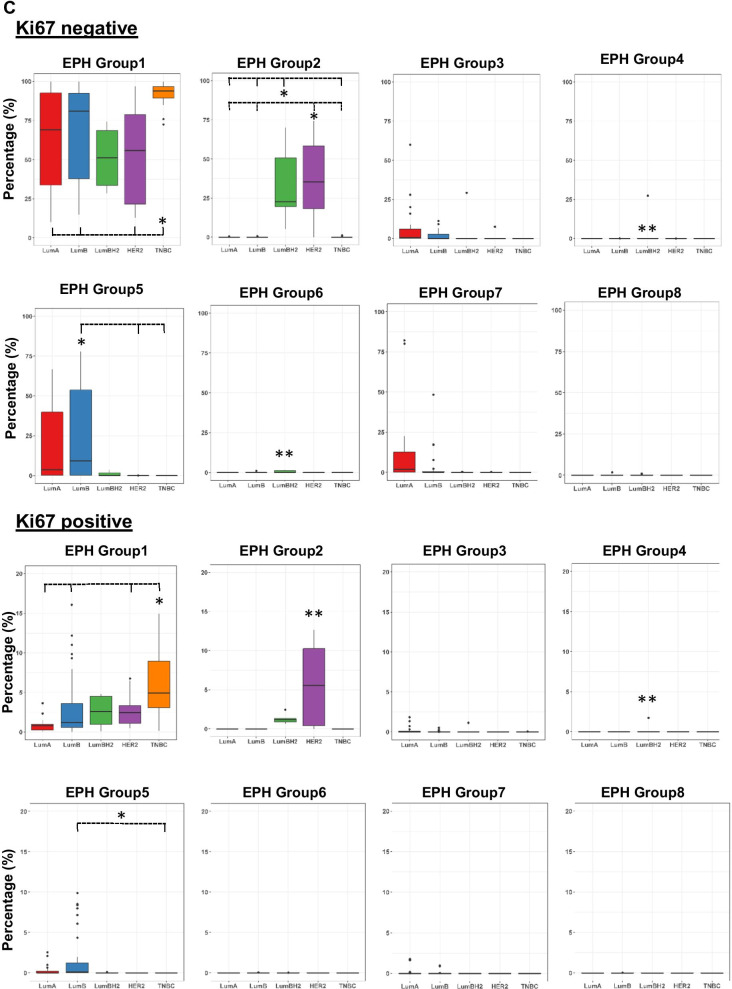
Abundance and distribution of EPH groups across IHC-surrogate subtypes of breast cancer. **A** Table showing the eight EPH groups of single cell classification according to thresholding of MxIF signal intensities of ER, PR and HER2. Ki67 positivity was also determined in each cell and denoted by shaded color bars. **B** Proportion of EPH groups (EPH1-8) in tumor cores that were classified as Luminal A-like (Number of cores, *N* = 24), Luminal B-like (HER2-) (*N* = 36), Luminal B-like (HER2+) (*N* = 6), HER2+ (non-luminal) (*N* = 13) and triple-negative (TNBC) (*N* = 22). Cores taken from the same cancer are aligned in pairs for comparison. Spot_032 and spot_059 are paired cores but assigned to different IHC-surrogate subtype and marked with asterisks (**). **C** Boxplot presentations of the percentages of cells of each EPH group that are either Ki67-negative (top panels) or Ki67-positive (bottom panels) across IHC subtypes. LumBH2- LumB, HER2+. To indicate subtypes that showed significant difference (*p* = 0.01) for a EPH group, the subtypes are marked with dashed lines with the major subtype denoted with an asterisk (*)—for EPH Group 1 (Ki67-), significant difference was found between TNBC and LumA, TNBC and LumB, and TNBC and LumBH2, respectively. Double asterisk (**) denotes the subtype that showed significant difference with all other subtypes—for EPH Group 2 (Ki67 +), significant difference were found between HER2 and LumA, HER2 and LumB, HER2 and LumBH2, and HER2 and TNBC

Cut-points for ER, PR and HER2 were applied to MxIF measured levels in each cell. As a result, 8 classes, which we refer to as EPH groups (for **E**R, **P**R and **H**ER2) emerged from the data and were color-labelled accordingly (Fig. 3A). Each group was further stratified as being Ki67+ or Ki67−. The abundance of EPH-classified cells in each TMA core was measured (Fig. 3B-C and Additional file 2: Table S1). Cores that were taken from the same cancer case were arranged together in pairs (Fig. 3B). Paired cores from all but one case were classified into the same IHC-surrogate subtype and generally showed similar distributions of EPH groups. That one case showed discordant classification, where one core was classified as Luminal A-like (LumA) (spot_032) and the second core as Luminal B-like (HER2-negative) (LumB) (spot_059). Luminal cancers were composed of varying proportions of cells from HER2-negative EPH groups (Groups 1, 3, 5 and 7) (Fig. 3B, C). LumB cancers harbored cells from PR-negative Groups 1 and 5, while LumA cancers also comprised PR+ Groups 3 and 7 cells. Only a few LumB, HER2+ (Luminal B-like, HER2+) cores were studied, and these mainly presented with cells from ER-weak, PR-negative EPH Group 1 and HER2+ Group 2. One particular LumB, HER2+ core (spot_117, Fig. 3B) showed composition of not only Groups 1 and 2, but also cells from ER-weak, PR-positive, HER2 ± Groups 3 and 4. Most of the cells in HER2+ (non-luminal) cases belonged to Groups 1 and 2 only (Fig. 3C). TNBC cases were predominately comprised of Group 1 cells (Fig. 3C).

We did not observe any strong association of Ki67 positivity to a single EPH group, but higher proliferative fractions were observed in Luminal B, HER2+ and TNBC cores, coinciding with EPH Groups 1, 2 and 5 (Fig. 3C), all of which are negative for PR.

Intra-tumoral heterogeneity was assessed by comparing the EPH composition and Ki67 positivity of single cells between cores taken from the same specimen. There were 41 cases with two cores studied. 6 out of 10 cases (60%) of LumA breast cancer showed significant statistical difference between cores, and 60% (9 out of 15 cases) of LumB cases showed significant differences. 1 out of 2 cases of LumB, HER2+, and all 4 HER2+ cases showed significant differences. As expected, only 20% (2 out of 10 cases) of TNBC showed significant differences between cores from the same specimen as most TNBC are composed of EPH Group 1 cells (Ki67+ or −) only.

### Co-expression patterns of Ki67, P53, P16 and P21 in IHC-surrogate subtypes reveals protein marker heterogeneity in TNBC

**Fig. 4:**
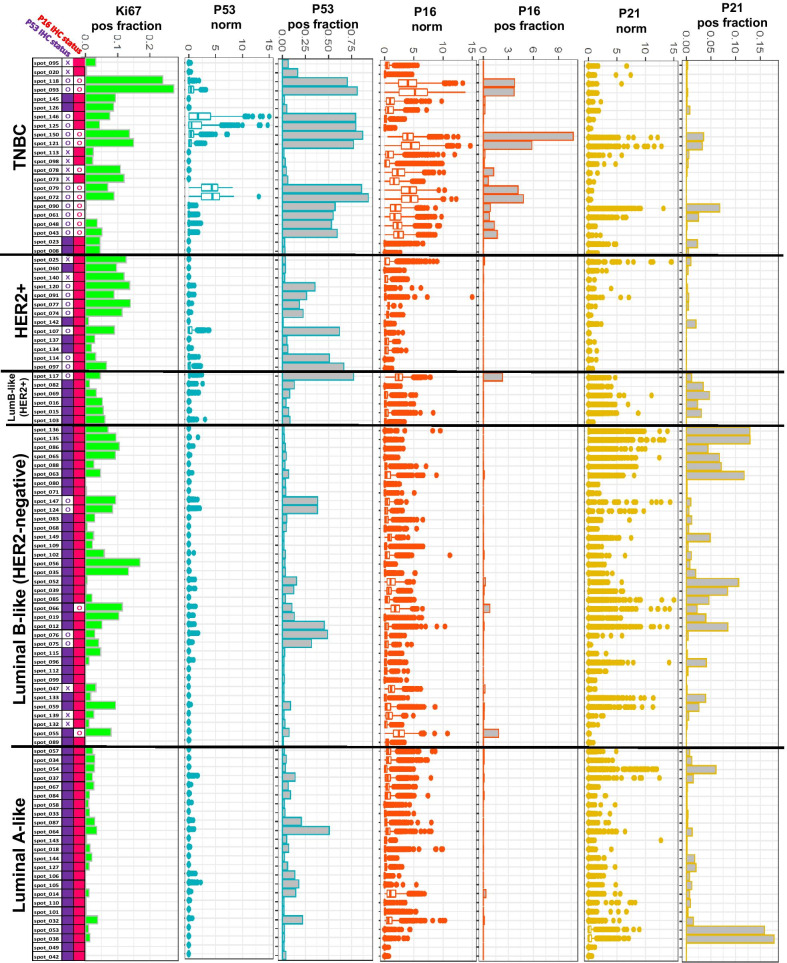
MxIF co-expression patterns of Ki67, P53, P21 and P16 in breast cancer cores with corresponding IHC evaluation. The fractions of MxIF-measured Ki67-positive cells (Ki67 pos fraction), P53-positive cells (P53 pos fraction, P16-positive cells (P16 pos fraction) and P21-positive cells (P21 pos fraction) per core, and the distribution of normalized MxIF signal intensities of P53 (P53norm), p16 (P16norm) and P21 (P21norm) in single cells are shown across all cores as classified by IHC-surrogate subtypes. The IHC status of P53 and P16 are also evaluated and presented with solid color box representing normal, “X” indicates the presence of null expression for P53 and “O” indicates the presence of overexpression of P53 or P16

Next, we analyzed the co-expression pattern of Ki67, P53, P21 and P16 in each core. Cores were arranged according to their IHC-surrogate subtypes (same order as Fig. 3B) to determine if specific signatures are more prevalent (Fig. 4). The normalized MxIF levels of P53, P16 and P21 per cell, as well as the positive fractions of Ki67, P53, P16 and P21 in each core are shown. Mutational status of P53 and P16 based on IHC evaluation are also shown for each case. We observed that proliferative fractions of MxIF-measured Ki67+ cells were high in some LumB, most HER2+ and most TNBC cases, with the latter exhibiting the highest levels. P53 and P16 aberrations were found most frequently in TNBC by IHC, either as null or as overexpression, as well as in HER2+ and a small fraction of LumB breast cancers. However, levels of P53 MxIF signal intensities were observed to be much higher in some TNBC tumors, compared to HER2+ or LumB cases which also exhibited P53 overexpression patterns in IHC. A large fraction of TNBC cases was also found to exhibit P16 overexpression by IHC or by high MxIF levels which coincided with IHC-scored P53 aberrations. On the other hand, two LumB cases which showed overexpression of P16 demonstrated normal P53 by IHC. P21 expression levels did not appear to associate with P53 or P16 expression patterns, yet a slightly higher proportion of LumB breast cancers, as compared to the other subtypes, demonstrated higher levels of P21 and P21-positive fractions measured by MxIF. The association between MxIF-measured P21 level and EPH Group classification was assessed and no significant dependence was observed (F-value = 0.003, *p-value* = 0.955).

### Neighborhood analysis to assess heterogeneity in cellular arrangement

**Fig. 5:**
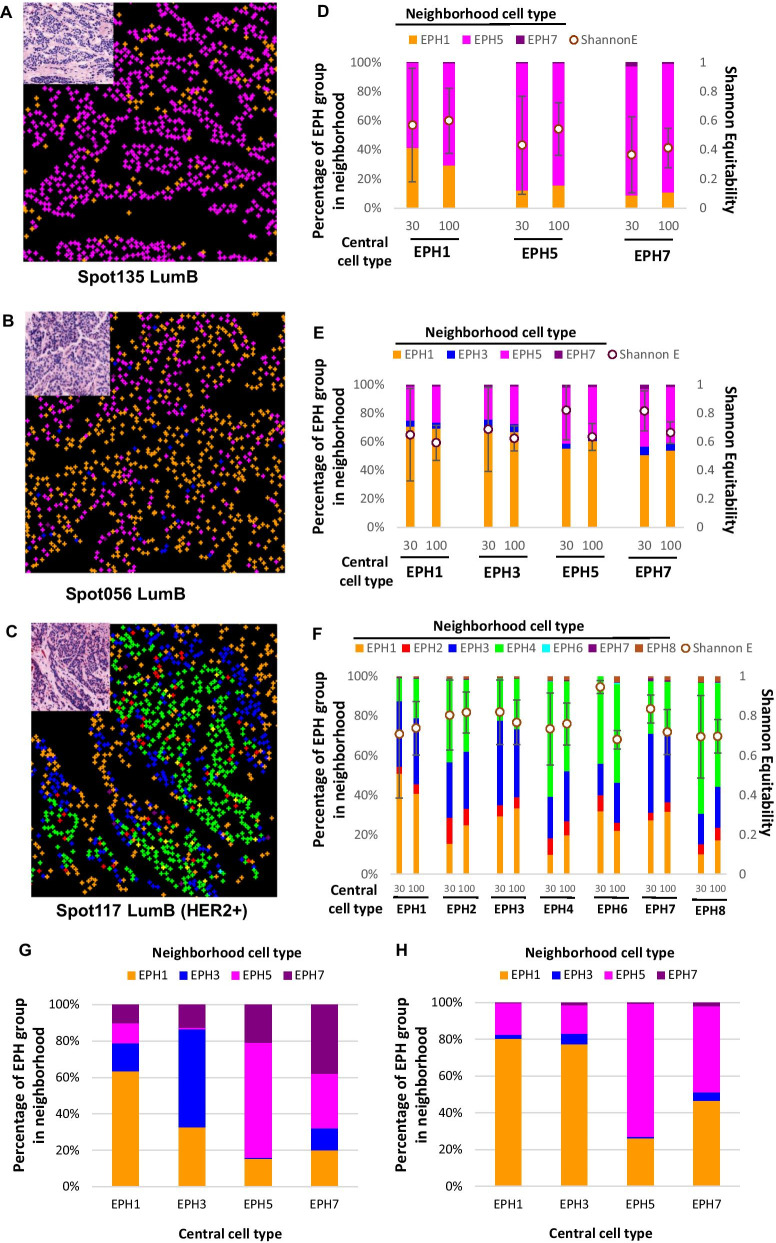
Spatial analysis of EPH groups. **A**–**C** The centroid location of each single cell is marked in color according to their EPH group assignment. The corresponding virtual H&E image is shown in inset. Two Luminal B-like cases (HER2-) and a Luminal B-like (HER2+) case are shown. **D**–**F** Quantitative evaluation of spatial heterogeneity. With neighborhood analysis, the percentages of neighboring EPH groups for each central EPH group are calculated for the indicated distance (30 or 100 mm in radius). The Shannon Equitability index for each central cell type is presented as the mean and standard deviation. **G**–**H** Cumulative analysis of the proportion of each EPH cell type in the neighborhood of the central cell type for **G** Luminal A-like cancers (*N* = 5) and for **H** Luminal B-like (HER2-negative) cancers (*N* = 5)

The spatial arrangement of cells with different protein co-expression signatures was studied. In Fig. 5 and Additional file 2: Fig. S1, the centroid of each cell, depicted by a color representing its EPH grouping was mapped back to its cellular location on the tissue section. Images from TMA cores of three LumB cases, one of which was also HER2+, are shown in Fig. 5. Spot135, a LumB (HER2-) tumor, illustrated a relatively homogeneous composition and consisted of predominately ERm/s EPH5 cells (Fig. 5A, D). Spot056, also a LumB (HER2-), appeared to be more heterogeneous in the composition of EPH groups and is composed of a majority of cells in EPH Groups 1 and 5, and a small number of cells in PR+ EPH Groups 3 and 7 (Fig. 5B, E). The LumB, HER2+ core (spot117) shown here was highly heterogeneous and composed of cells from all groups except for Group 5 (Fig. 5C, F). Here, cells from the two predominant groups (EPH3 and 4) appeared to form spatial clusters within their own group.

Quantitative analysis of heterogeneity in cellular arrangement with Shannon Equitability Index (E_H_) is illustrated in Fig. 5D–F. E_H_ was calculated for each (central) cell for the indicated size of neighborhood (30 and 100 μm in radii) and presented as the mean for each EPH cell type. In spot135, E_H_ was found to be significantly higher for central EPH1 cells in both 30 and 100 μm neighborhoods (*p* < 0.01) (Fig. 5D), suggesting that EPH Group 1 cells tend to have more heterogeneous mix of cells in their proximity compared to other EPH groups in this cancer. In the other LumB case (spot056), E_H_ measured for EPH5 central cells was significantly higher than EPH Groups 1 and 3 in the 30 μm neighborhood (*p* < 0.01) (Fig. 5E). In the LumB, HER2+ case examined (spot117), E_H_ evaluated for central cells of both EPH Groups 2 and 3 was significantly higher than for EPH1 and 4 (*p* < 0.01), suggesting that EPH2 and 3 cells are more likely to be present in a heterogeneous cellular environment compared to other cellular groups in this tumor (Fig. 5F).

To determine if the neighborhood environment for certain EPH Group cells demonstrate particular patterns, we studied the cumulative neighboring cell types of central EPH cell type in 5 LumA and 5 LumB cores (Fig. 5G, H). For LumA cores (Fig. 5G), each EPH group that was present appeared to have a higher likelihood to being in close proximity to cells of the same EPH group. For LumB cores (Fig. 5H), only small numbers of EPH3 and 7 cells were present, while EPH1 and 5 cells tended to be most likely to cluster with their own group.

### Quantification of immune cells and their spatial organization

**Fig. 6:**
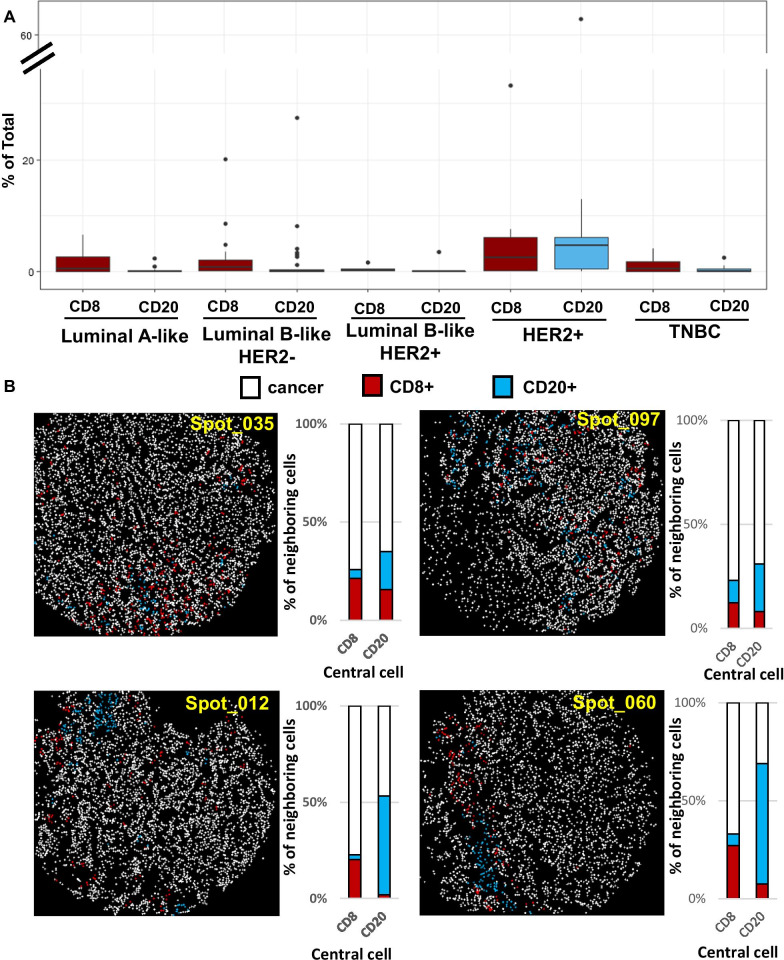
Spatial analysis of CD8 T-lymphocytes and CD20 B-lymphocytes. **A** Quantification of CD8 and CD20 lymphocytes presented as percentages of total cells in cancers categorized by IHC subtypes. Number of cores included in LumA (*N* = 24), LumB (*N* = 36), LumB, HER2+ (*N* = 6), HER2 (*N* = 13), TNBC (*N* = 22). **B** Quantitative evaluation of spatial arrangement. All cells in the core were classified as cancer, CD8 or CD20 lymphocytes. With neighborhood analysis, the percentages of neighboring cell types within 100 mm for each central CD8 or CD20 were quantified

In addition to analysis of breast cancer cells, densities of CD8 T-lymphocytes and CD20 B-lymphocytes in the tumor microenvironment were quantified. We found higher levels of both CD8 and CD20 lymphocytes in the HER2+ cancers compared to the other IHC subtypes (Fig. 6A). Next we attempted to evaluate the spatial localization patterns of these lymphocytes using co-occurrence matrices. Four representative cases with similar densities of CD8 (3.5–8.6%) and CD20 (3.3–8.2%) are shown in Fig. 6B. The localization maps indicate that the lymphocytes in spots 035 and 097 have more spatially scattered, tumor-infiltrating patterns, whereas in spots 012 and 060 lymphocytes tended to be arranged in close proximity, particularly for the CD20 B-lymphocytes. These observations were also apparent from the quantification of percentage of neighboring cells for each central CD8 or CD20 cell. While the majority of neighboring cells for CD8 and CD20 were PCK+ cancer cells in spots 035 and 097, about half of neighboring cells for CD20 were also CD20 cells in spots 012 and 060. These findings suggest that cancers that showed similar levels of immune densities could exhibit heterogeneous spatial patterns.

## Discussion

Breast cancers are traditionally classified based on morphology including histologic grade and the expressions of ER, PR and HER2 following established guidelines. The St. Gallen International Expert Consensus on the primary therapy of early breast cancers have established the use of IHC staining to define surrogates for molecularly defined intrinsic subtypes [30]. In this proof of principle study, we have reported the use of protein marker multiplexing with MxIF to examine protein biomarker co-expression patterns of single cells within each IHC-surrogate subtype. Comparison of the distribution of MxIF intensity levels of each marker and the corresponding IHC scoring illustrated that MxIF-measured expression correlates well overall with IHC classifications. When individual cells were characterized by their MxIF-measured levels of ER, PR and HER2, they segregated into groups to which we refer here as EPH, and demonstrated marked heterogeneity between cores within the Luminal A/B-like St. Gallen IHC subtypes. Heterogeneity was also observed in TNBC cancers with varying P53 and P16 levels. P53 protein levels measured with MxIF were much higher in some TNBC cancers, compared to HER2+ or luminal B-like cases which also harbored P53 overexpression identified with IHC. Our study illustrates that heterogeneity within each St. Gallen IHC-surrogate subtype could be further explained by quantitative analysis of co-expression signatures of key biomarkers. Addition of these refined descriptors could assist in the characterization of individual breast cancer to improve management and long-term prognostication of the disease.

When single cells were grouped according to the co-expression signatures of ER, PR and HER2, we found that significant levels of variation exist within ER+ cancers, especially in the proportion and staining intensities of ER and PR. Currently, depending on the patient’s menopausal status, endocrine therapy is recommended for hormone receptor-positive, HER2-negative cancers, defined as ER/PR > 1%. Recent recommendations from the American Society of Clinical Oncology/College of American Pathologists (ASCO/CAP) for ER and PR test reporting include the introduction of a new category of “ER Low Positive” in light of limited data on the benefit of endocrine therapy for cancers with 1% to 10% of ER+ cells [35]. Improved characterization of cancers with low to intermediate range of hormonal receptor expression will help to identify subgroups of cases with poor prognosis which may require more aggressive treatment or monitoring. Hormonal receptor-positive, HER2-positive breast cancers, which constitute 5% of all breast cancers, also exhibit high degrees of heterogeneity in ER and HER2 expression which has been shown to be associated with their response to neoadjuvant chemotherapy and HER2-targeted therapy [36]. Our analysis included only a few Luminal B-like, HER2+ cancers. Interestingly, we found as reported by others [37, 38] that cells co-expressing high levels of both ER and HER2 are rare, while co-expression of PR and HER2 was detected in a small fraction of cells. Ki67 positivity appeared to co-localize with EPH groups that are PR-negative. This observation aligns with previous findings which suggest that the loss of, or low values of PR expression is an adverse prognostic factor [39, 40].

Spatial arrangement of cells was evaluated quantitatively by measuring the frequency of each cell type in a user-defined distance from the central cell type. Heterogeneity in cellular neighborhood was also quantified by calculating the Shannon Equitability index for each central cell. Although only a few cases were presented here, certain EPH groups appear to be localized in neighborhoods that were less heterogeneous. Since our limited dataset lacks outcome information of the cases studied, the clinical significance of these findings is not clear at this point. Nevertheless we believe that this tool for quantifying cellular arrangements could be useful when combined with other molecular profiling assays, particularly in the study of mutational evolution. Cells harboring potential driver mutations will exhibit higher proliferative capacity, resulting in these cells being closely arranged in cellular homogeneous neighborhoods. Such cellular neighborhood analysis could also be used to predict functional interaction between cell groups, such as the relationships between infiltrating immune subsets or between immune and cancer cells. We demonstrated that between cancers that have similar levels of CD8 and CD20 lymphocytes, some cases showed a more scattered infiltration pattern, while in others the lymphocytes were arranged in close proximity. However, the limited size of the TMA cores studied here prevented us from properly assessing immune densities and localization patterns over the entire lesion as would be done in the pathology lab [41]. Nevertheless, we believe these methods, when applied to whole tissue sections, could be useful for evaluating response to immunotherapy, where the type, density and localization of immune subsets have shown to predict long-term outcomes and response to therapy [41–47].

Our current analysis was based on gating for cancer cells on the basis of larger sizes compared to normal epithelial cells in perimeter and area in pixels. Selection of cancer cells was further enriched after thresholding of PCK26 level in luminal and HER2+ cancers. TNBC cells express weak PCK26, preventing the use of a threshold for gating. Therefore, cancer cells were manually annotated in the TNBC cancers. Nevertheless, “contamination” with non-cancer cells in our study population was inevitable and these could be tumor-infiltrating lymphocytes, fibroblasts or vascular cells. Another technical challenge is the low sensitivity in calibrating the MxIF measurement in distinguishing ER weakly positive cells from ER negative cells. A more granular calibration study, with the goal of improving the correlation of MxIF measurements to IHC histoscores in breast cancer is being conducted to develop methods that could translate multiplex-defined signatures to clinical subtypes.

Recent advances in high-dimensional proteomics technologies have allowed the study of breast cancer with hundreds or even thousands of protein markers and revealed the presence of a myriad of cellular phenotypes in the cancer and the tumor microenvironment that may further define each PAM50 intrinsic molecular subtype [48–52]. Although our analysis here is limited to seven markers, our comparison with IHC-surrogate subtypes and IHC staining patterns illustrate how the MxIF co-expression signatures could be translated to phenotypes identified in the clinical setting. We believe that a detailed quantitative assessment of protein markers co-expression on single cells, in the context of intensity grading and spatial arrangement, could improve the precision in stratifying the disease for treatment assignment and monitoring. To this goal, we are applying the methods reported here to study whole tissue sections from a cohort of whole-mount processed breast lumpectomies and correlating with clinical outcome information. A combined evaluation with molecular analyses such as targeted mutational or RNA profiling, will augment our protein signature and clustering analysis. The ability to study spatial arrangement of cell groups, particularly between cancer cells and cells in the stroma and the heterogeneity of such arrangement could provide valuable insight into improved management of breast cancer.

## Conclusions

We report on a proof-of-concept study where single-cell image analysis of protein multiplexing is used to assess heterogeneity within IHC-defined breast cancer subtypes. Biomarker expression signatures evaluated on single cells, revealed heterogeneous composition and spatial arrangement of subgroups. Furthermore, cancers with the same IHC score of overexpression of P53 and/or P16 exhibited a range of expression levels when measured in individual cells. These findings motivate a more refined stratification of breast cancers based on the expression levels of protein biomarkers and evaluation of correlation with outcomes. This information may improve the characterization of breast cancers and predicting their responsiveness to therapy.

## Supplementary Information

**Additional file 1**. Cheung_BrCaMxIF_RawDataAllCells.csv. This file contains the raw MxIF data output from all single cells of the TMA analysed.

**Additional file 2**. Figure and Table.

## Data Availability

The dataset consisting of raw output of the MxIF study is included as an additional file to this submission—“Cheung_BrCaMxIF_RawDataAllCells.csv”. It will be placed in a public repository before this article is published.

## Abbreviations

ANOVA: Analysis of variance
ASCO/CAP: American Society of Clinical Oncology/College of American Pathologists
CDK: Cyclin-dependent kinase
Cox2: Cyclooxygenase-II
E_H_: Shannon Equitability Index
EPH: **E**R/**P**R/**H**ER2 (group)
ER: Estrogen Receptor
FFPE: Formalin-fixed paraffin-embedded
FISH: Fluorescent in situ hybridisation
HER2: Epidermal Growth Factor Receptor 2
IHC: Immunohistochemistry
LumA: Luminal A-like subtype
LumB: Luminal B-like (HER2-negative) subtype
LumB, HER2+: Luminal B-like (HER2-positive) subtype
HER2+: HER2-positive (non-luminal) subtype
m/s: Moderately or strongly stained
MxIF: Multiplex in Immunofluorescence
O/E: Over-expression
PR: Progesterone Receptor
TMA: Tissue micro-array
TNBC: Triple-negative breast cancer
Tukey’s HSD: Tukey’s honestly significant difference
vH&E: Virtual Hematoxylin & Eosin

## References

[1] Koboldt DC, Fulton RS, McLellan MD, Schmidt H, Kalicki-Veizer J, McMichael JF (2012). Comprehensive molecular portraits of human breast tumours. Nature.

[2] Perou CM, Sørlie T, Eisen MB, van de Rijn M, Jeffrey SS, Rees CA (2000). Molecular portraits of human breast tumours. Nature.

[3] Cheang MCU, Martin M, Nielsen TO, Prat A, Voduc D, Rodriguez-Lescure A (2015). Defining breast cancer intrinsic subtypes by quantitative receptor expression. Oncologist.

[4] Ali HR, Rueda OM, Chin S-F, Curtis C, Dunning MJ, Aparicio SA (2014). Genome-driven integrated classification of breast cancer validated in over 7,500 samples. Genome Biol.

[5] Curtis C, Shah SP, Chin S-F, Turashvili G, Rueda OM, Dunning MJ (2012). The genomic and transcriptomic architecture of 2,000 breast tumours reveals novel subgroups. Nature.

[6] Russnes HG, Lingjærde OC, Børresen-Dale A-L, Caldas C, Nord S, Caldas C (2017). Breast cancer molecular stratification- from intrinsic subtypes to integrative clusters. Am J Pathol.

[7] Dawson S-J, Rueda OM, Aparicio S, Caldas C (2013). A new genome-driven integrated classification of breast cancer and its implications. EMBO J.

[8] Goldhirsch A, Winer EP, Coates AS, Gelber RD, Piccart-Gebhart M, Thürlimann B, et al. Personalizing the treatment of women with early breast cancer: highlights of the St Gallen International Expert Consensus on the Primary Therapy of Early Breast Cancer 2013. 2013

[9] Curigliano G, Burstein HJ, Winer EP, Gnant M, Dubsky P, Loibl S (2017). De-escalating and escalating treatments for early-stage breast cancer: The St. Gallen International Expert Consensus Conference on the Primary Therapy of Early Breast Cancer 2017. Ann Oncol.

[10] Bastien RR, Rodríguez-Lescure Á, Ebbert MT, Prat A, Munárriz B, Rowe L (2012). PAM50 Breast cancer subtyping by RT-qPCR and concordance with standard clinical molecular markers. BMC Med Genomics.

[11] Fernandez-Martinez A, Pascual T, Perrone G, Morales S, de la Haba J, González-Rivera M (2017). Limitations in predicting PAM50 intrinsic subtype and risk of relapse score with Ki67 in estrogen receptor-positive HER2- negative breast cancer. Oncotarget.

[12] Liu MC, Pitcher BN, Mardis ER, Davies SR, Friedman PN, Snider JE (2016). PAM50 gene signatures and breast cancer prognosis with adjuvant anthracycline-and taxane-based chemotherapy: Correlative analysis of C9741 (alliance). Npj Breast Cancer..

[13] Prat A, Cheang MCU, Galván P, Nuciforo P, Paré L, Adamo B (2016). Prognostic value of intrinsic subtypes in hormone receptor-positive metastatic breast cancer treated with letrozole with or without lapatinib. JAMA Oncol.

[14] Prat A, Fan C, Fernández A, Hoadley KA, Martinello R, Vidal M (2015). Response and survival of breast cancer intrinsic subtypes following multi-agent neoadjuvant chemotherapy. BMC Med..

[15] Pogue-Geile KL, Song N, Jeong JH, Gavin PG, Kim SR, Blackmon NL (2015). Intrinsic subtypes, PIK3CA mutation, and the degree of benefit from adjuvant trastuzumab in the NSABP B-31 trial. J Clin Oncol.

[16] Nielsen TO, Parker JS, Leung S, Voduc D, Ebbert M, Vickery T (2010). A comparison of PAM50 intrinsic subtyping with immunohistochemistry and clinical prognostic factors in tamoxifen-treated estrogen receptor-positive breast cancer. Clin Cancer Res.

[17] Szymiczek A, Lone A, Akbari MR (2020). Molecular intrinsic versus clinical subtyping in breast cancer: a comprehensive review. Clinical genetics.

[18] Rueda OM, Sammut SJ, Seoane JA, Chin SF, Caswell-Jin JL, Callari M (2019). Dynamics of breast-cancer relapse reveal late-recurring ER-positive genomic subgroups. Nature.

[19] Burstein MD, Tsimelzon A, Poage GM, Covington KR, Contreras A, Fuqua SAW (2015). Comprehensive genomic analysis identifies novel subtypes and targets of triple-negative breast cancer. Clin Cancer Res.

[20] Echavarria I, Lopez-Tarruella S, Picornell A, García-Saenz JA, Jerez Y, Hoadley K (2018). Pathological response in a triple-negative breast cancer cohort treated with neoadjuvant carboplatin and docetaxel according to Lehmann’s refined classification. Clin Cancer Res.

[21] Gingras I, Gebhart G, De Azambuja E, Piccart-Gebhart M. HER2-positive breast cancer is lost in translation: time for patient-centered research. Vol. 14, Nature Reviews Clinical Oncology. Nature Publishing Group; 2017. p. 669–81. 10.1038/nrclinonc.2017.96 28762384

[22] Karaayvaz M, Cristea S, Gillespie SM, Patel AP, Mylvaganam R, Luo CC (2018). Unravelling subclonal heterogeneity and aggressive disease states in TNBC through single-cell RNA-seq. Nat Commun..

[23] Lehmann BD, Jovanović B, Chen X, Estrada MV, Johnson KN, Shyr Y (2016). Refinement of triple-negative breast cancer molecular subtypes: Implications for neoadjuvant chemotherapy selection. PLoS ONE..

[24] Carey LA, Berry DA, Cirrincione CT, Barry WT, Pitcher BN, Harris LN (2016). Molecular heterogeneity and response to neoadjuvant human epidermal growth factor receptor 2 targeting in CALGB 40601, a randomized phase III trial of paclitaxel plus trastuzumab with or without lapatinib. J Clin Oncol.

[25] Whitworth P, Beitsch P, Mislowsky A, Pellicane JV, Nash C, Murray M (2017). Chemosensitivity and endocrine sensitivity in clinical luminal breast cancer patients in the prospective neoadjuvant breast registry symphony trial (NBRST) predicted by molecular subtyping. Ann Surg Oncol.

[26] Lambertini M, Campbell C, Gelber RD, Viale G, McCullough A, Hilbers F, Korde LA, Werner O, Chumsri S, Jackisch C, Wolff AC, Vaz-Luis I, Ferreira AR, Prat A, Moreno-Aspitia A, Piccart M, Loi S, de Azambuja E (2019). Dissecting the effect of hormone receptor status in patients with HER2-positive early breast cancer: exploratory analysis from the ALTTO (BIG 2–06) randomized clinical trial. Breast Cancer Res Treat..

[27] Gerdes MJ, Sevinsky CJ, Sood A, Adak S, Bello MO, Bordwell A (2013). Highly multiplexed single-cell analysis of formalin-fixed, paraffin-embedded cancer tissue. Proc Natl Acad Sci.

[28] Fitzgibbons PL, Murphy DA, Hammond MEH, Allred DC, Valenstein PN (2010). Recommendations for validating estrogen and progesterone receptor immunohistochemistry assays. Arch Pathol Lab Med.

[29] Wolff AC, Hammond MEH, Hicks DG, Dowsett M, McShane LM, Allison KH (2013). Recommendations for human epidermal growth factor receptor 2 testing in breast cancer: American Society of Clinical Oncology/College of American Pathologists clinical practice guideline update. J Clin Oncol.

[30] Goldhirsch A, Winer EP, Coates AS, Gelber RD, Piccart-Gebhart M, Thürlimann B (2013). Personalizing the treatment of women with early breast cancer: highlights of the St Gallen International Expert Consensus on the Primary Therapy of Early Breast Cancer 2013. Ann Oncol.

[31] Gerdes MJ, Sood A, Montalto MC, Can A, Ginty F, Bresnahan MA, Filkins RJ PZ. Sequential analysis of biological samplese. 2010. p. US7741045.

[32] Magurran AE (1988). Ecological diversity and its measurement. Ecological diversity and its measurement.

[33] Ali Hashmi A, Naz S, Hashmi SK, Hussain ZF, Irfan M, Khan EY (2018). Prognostic significance of p16 & p53 immunohistochemical expression in triple negative breast cancer 11 Medical and Health Sciences 1112 Oncology and Carcinogenesis. BMC Clin Pathol..

[34] Yemelyanova A, Vang R, Kshirsagar M, Lu D, Marks MA, Shih IM (2011). Immunohistochemical staining patterns of p53 can serve as a surrogate marker for TP53 mutations in ovarian carcinoma: an immunohistochemical and nucleotide sequencing analysis. Mod Pathol.

[35] Allison KH, Hammond MEH, Dowsett M, McKernin SE, Carey LA, Fitzgibbons PL (2020). Estrogen and progesterone receptor testing in breast cancer: American society of clinical oncology/college of American pathologists guideline update. Arch Pathol Lab Med.

[36] Rye IH, Trinh A, Sætersdal AB, Nebdal D, Lingjærde OC, Almendro V (2018). Intratumor heterogeneity defines treatment-resistant HER2+ breast tumors. Mol Oncol.

[37] Chen C, Peng J, Xia H, Wu Q, Zeng L, Xu H (2010). Quantum-dot-based immunofluorescent imaging of HER2 and ER provides new insights into breast cancer heterogeneity. Nanotechnology..

[38] Giltnane JM, Moeder CB, Camp RL, Rimm DL (2009). Quantitative multiplexed analysis of ErbB family coexpression for primary breast cancer prognosis in a large retrospective cohort. Cancer.

[39] Chen S, Huang L, Chen CM, Shao ZM (2015). Progesterone receptor loss identifies luminal-type local advanced breast cancer with poor survival in patients who fail to achieve a pathological complete response to neoadjuvant chemotherapy. Oncotarget.

[40] Prat A, Cheang MCU, Martín M, Parker JS, Carrasco E, Caballero R (2013). Prognostic significance of progesterone receptor-positive tumor cells within immunohistochemically defined luminal a breast cancer. J Clin Oncol.

[41] Salgado R, Denkert C, Demaria S, Sirtaine N, Klauschen F, Pruneri G (2015). The evaluation of tumor-infiltrating lymphocytes (TILs) in breast cancer: recommendations by an International TILs Working Group 2014. Ann Oncol.

[42] Denkert C, Von Minckwitz G, Brase JC, Sinn BV, Gade S, Kronenwett R (2015). Tumor-infiltrating lymphocytes and response to neoadjuvant chemotherapy with or without carboplatin in human epidermal growth factor receptor 2-positive and triple-negative primary breast cancers. J Clin Oncol.

[43] Heppner BI, Untch M, Denkert C, Pfitzner BM, Lederer B, Schmitt W (2016). Tumor-infiltrating lymphocytes: a predictive and prognostic biomarker in neoadjuvant-treated HER2-positive breast cancer. Clin Cancer Res.

[44] Heindl A, Sestak I, Naidoo K, Cuzick J, Dowsett M, Yuan Y (2017). Relevance of spatial heterogeneity of immune infiltration for predicting risk of recurrence after endocrine therapy of ER+ breast cancer. J Natl Cancer Inst..

[45] Karn T, Denkert C, Weber KE, Holtrich U, Hanusch C, Sinn BV (2020). Tumor mutational burden and immune infiltration as independent predictors of response to neoadjuvant immune checkpoint inhibition in early TNBC in GeparNuevo. Ann Oncol.

[46] Maley CC, Koelble K, Natrajan R, Aktipis A, Yuan Y (2015). An ecological measure of immune-cancer colocalization as a prognostic factor for breast cancer. Breast Cancer Res.

[47] Zhang AW, McPherson A, Milne K, Kroeger DR, Hamilton PT, Miranda A (2018). Interfaces of malignant and immunologic clonal dynamics in ovarian cancer. Cell.

[48] Angelo M, Bendall SC, Finck R, Hale MB, Hitzman C, Borowsky AD (2014). Multiplexed ion beam imaging of human breast tumors. Nat Med.

[49] Johansson HJ, Socciarelli F, Vacanti NM, Haugen MH, Zhu Y, Siavelis I, Fernandez-Woodbridge A, Aure MR, Sennblad B, Vesterlund M, Branca RM, Orre LM, Huss M, Fredlund E, Beraki E, Garred Ø, Boekel J, Sauer T, Zhao W, Nord S, Hoglander EK, Jans DC, Brismar H, Haukaas TH, Bathen TF, Schlichting E, Naume B, Luders T, Borgen E, Kristensen VN, Russnes HG, Lingjærde OC, Mills GB, Sahlberg KK, Børresen-Dale A-L, Lehtiö J (2019). Breast cancer quantitative proteome and proteogenomic landscape. Nat Commun..

[50] McCart Reed AE, Bennett J, Kutasovic JR, Kalaw E, Ferguson K, Yeong J (2020). Digital spatial profiling application in breast cancer: a user’s perspective. Virchows Arch.

[51] Ali HR, Jackson HW, Zanotelli VRT, Danenberg E, Fischer JR, Bardwell H (2020). Imaging mass cytometry and multiplatform genomics define the phenogenomic landscape of breast cancer. Nat Cancer.

[52] Jackson HW, Fischer JR, Zanotelli VRT, Ali HR, Mechera R, Soysal SD (2020). The single-cell pathology landscape of breast cancer. Nature.

