# Supplementary material for: Quantitative single-cell analysis of immunofluorescence protein multiplex images illustrates biomarker spatial heterogeneity within breast cancer subtypes
Source: Breast Cancer Res. 2021 Dec 18;23:114. doi: 10.1186/s13058-021-01475-y (PMC8684264; doi:10.1186/s13058-021-01475-y)
Supplement: Supplementary file 2 — Additional file 2. Figure and Table. [file 13058_2021_1475_MOESM2_ESM.pdf]

Quantitative single-cell analysis of immunofluorescence protein multiplex images illustrates biomarker spatial heterogeneity within breast cancer subtypes

Alison Min-Yan Cheung, Dan Wang, Kela Liu, Tyna Hope, Mayan Murray, Fiona Ginty, Sharon Nofech-Mozes, Anne Louise Martel & Martin Joel Yaffe

Additional File #2

EPH group	E-/w<br>P-H-K-<br>1a	E-/w<br>P-H-K+<br>1b	E-/w<br>P-H+K-<br>2a	E-/w<br>P-H+K+<br>2b	E-/w<br>P+H-K-<br>3a	E-/w<br>P+H-K+<br>3b	E/w<br>P+H+K-<br>4a	E/w<br>P+H+K+<br>4b	Em/s<br>P-H-K-<br>5a	Em/s<br>P-H-K+<br>5b	Em/s<br>P-H+K-<br>6a	Em/s<br>P-H+K+<br>6b	Em/s<br>P+H-K-<br>7a	Em/s<br>P+H-K+<br>7b	Em/s<br>P+H+K-<br>8a	Em/s<br>P+H+K+<br>8b
**IHC-subtype**																
**LumA (N=24)**																
min	0.100	0.000	0.000	0.000	0.000	0.000	0.000	0.000	0.000	0.000	0.000	0.000	0.000	0.000	0.000	0.000
max	0.999	0.036	0.005	0.000	0.600	0.018	0.000	0.000	0.667	0.026	0.000	0.000	0.822	0.018	0.000	0.000
mean	0.626	0.008	0.000	0.000	0.067	0.002	0.000	0.000	0.176	0.003	0.000	0.000	0.116	0.002	0.000	0.000
std.dev	0.316	0.008	0.001	0.000	0.135	0.005	0.000	0.000	0.239	0.007	0.000	0.000	0.227	0.005	0.000	0.000
**LumB (N=36)**																
min	0.147	0.000	0.000	0.000	0.000	0.000	0.000	0.000	0.000	0.000	0.000	0.000	0.000	0.000	0.000	0.000
max	0.997	0.161	0.007	0.000	0.112	0.005	0.001	0.000	0.777	0.099	0.010	0.000	0.484	0.010	0.018	0.000
mean	0.661	0.030	0.000	0.000	0.016	0.000	0.000	0.000	0.246	0.017	0.000	0.000	0.028	0.001	0.000	0.000
std.dev	0.301	0.040	0.001	0.000	0.028	0.001	0.000	0.000	0.284	0.030	0.002	0.000	0.088	0.002	0.003	0.000
**LumB HER2+ (N=6)**																
min	0.285	0.001	0.053	0.007	0.000	0.000	0.000	0.000	0.000	0.000	0.000	0.000	0.000	0.000	0.000	0.000
max	0.742	0.048	0.700	0.024	0.291	0.011	0.274	0.018	0.037	0.001	0.016	0.000	0.004	0.000	0.009	0.000
mean	0.512	0.026	0.332	0.013	0.049	0.002	0.046	0.003	0.010	0.000	0.006	0.000	0.001	0.000	0.002	0.000
std.dev	0.207	0.021	0.254	0.006	0.119	0.005	0.112	0.007	0.016	0.000	0.008	0.000	0.002	0.000	0.004	0.000
**HER2 (N=13)**																
min	0.130	0.005	0.001	0.000	0.000	0.000	0.000	0.000	0.000	0.000	0.000	0.000	0.000	0.000	0.000	0.000
max	0.968	0.068	0.743	0.126	0.076	0.000	0.001	0.000	0.001	0.000	0.000	0.000	0.003	0.000	0.000	0.000
mean	0.530	0.028	0.382	0.054	0.006	0.000	0.000	0.000	0.000	0.000	0.000	0.000	0.000	0.000	0.000	0.000
std.dev	0.311	0.020	0.269	0.050	0.021	0.000	0.000	0.000	0.000	0.000	0.000	0.000	0.001	0.000	0.000	0.000
**TNBC (N=22)**																
min	0.725	0.001	0.000	0.000	0.000	0.000	0.000	0.000	0.000	0.000	0.000	0.000	0.000	0.000	0.000	0.000
max	0.999	0.275	0.012	0.000	0.000	0.000	0.000	0.000	0.000	0.000	0.000	0.000	0.000	0.000	0.000	0.000
mean	0.919	0.080	0.001	0.000	0.000	0.000	0.000	0.000	0.000	0.000	0.000	0.000	0.000	0.000	0.000	0.000
std.dev	0.072	0.072	0.003	0.000	0.000	0.000	0.000	0.000	0.000	0.000	0.000	0.000	0.000	0.000	0.000	0.000

Table S1: Summary statistics of the proportions of each EPH group (1a-8b) in TMA cores as classified based on IHC-surrogate subtypes. The expression pattern of Estrogen Receptor (E) as negative to weak (-/w) or moderate to strong (m/s), of Progesterone Receptor (P), HER2 (H) and Ki67 (K) as positive/negative (+/-) are indicated. The number of cores in each subtype is as indicated (N).

A

EPH	ER	PR	HER2	
1	-/w	-	-	
2	-/w	-	+	
3	-/w	+	-	
4	-/w	+	+	
5	m/s	-	-	
6	m/s	-	+	
7	m/s	+	-	
8	m/s	+	+	

B

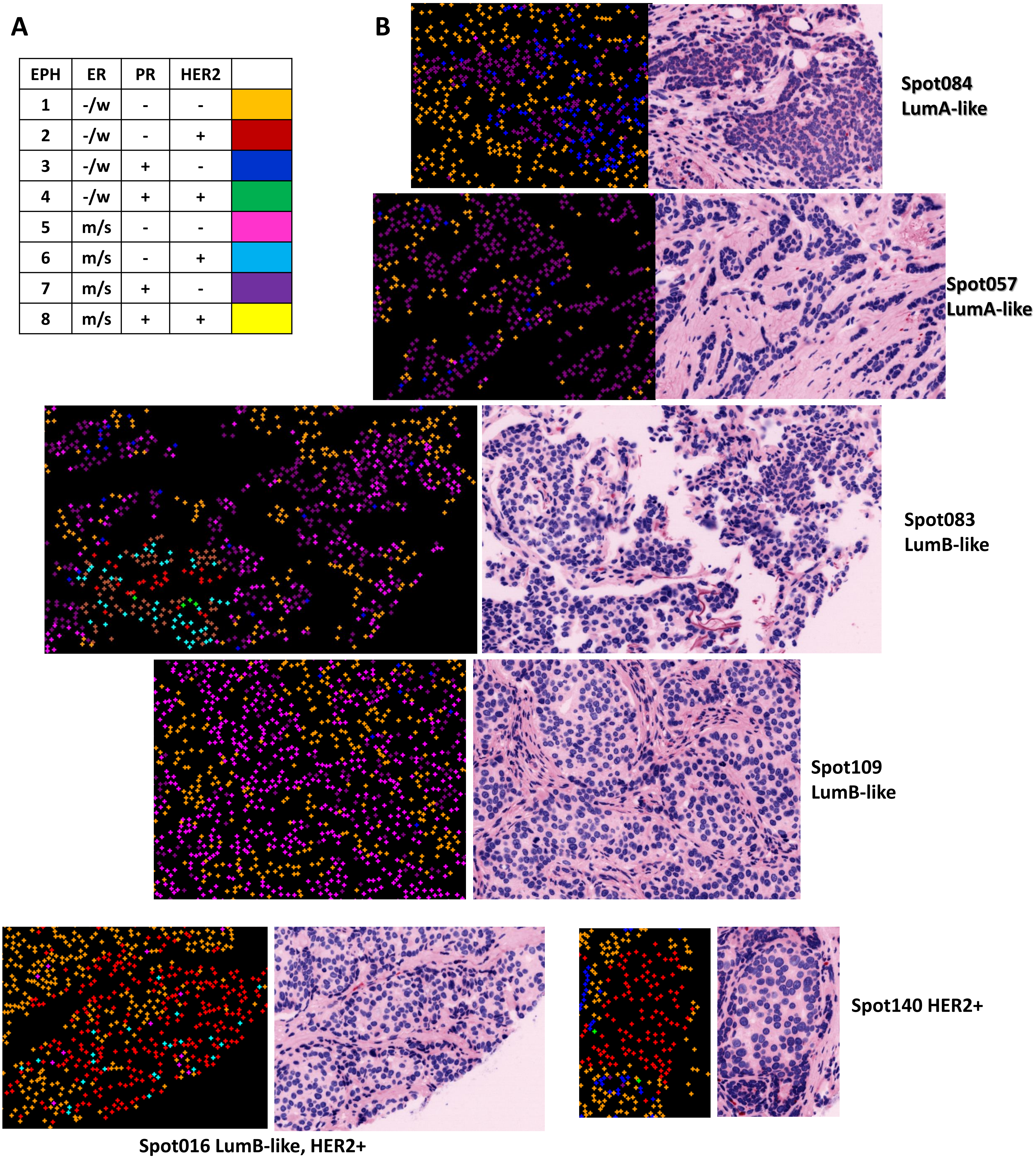

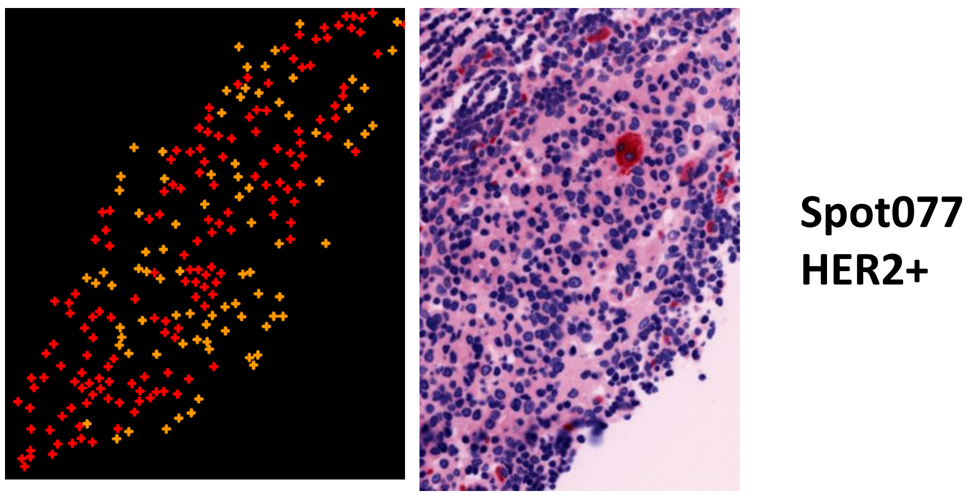

Figure S1. Additional representative examples of single cells classified to EPH groups and mapped to their cellular locations. (A) EPH groups and color code. (B) Single cells mapped to their EPH groups from 7 representative spots and their corresponding IHC-subtype. The vH&E of the same region is also shown.
